# Precondensed Plasmid DNA Enhances CAR‑T Cell Generation via Lipid Nanoparticles

**DOI:** 10.1021/acsomega.5c04308

**Published:** 2025-07-04

**Authors:** Andrea Pirrottina, Serena Renzi, Luca Digiacomo, Francesca Giulimondi, Valentina De Lorenzi, Samuele Ghignoli, Luca Pesce, Francesco Cardarelli, Francesco Mura, Giacomo Parisi, Luca Buccini, Chiara Cassone, Alessandra Zingoni, Daniela Pozzi, Giulio Caracciolo

**Affiliations:** † Department of Molecular Medicine, Sapienza University of Rome, Rome 00161, Italy; ‡ Laboratorio NEST, 19004 Scuola Normale Superiore, Pisa 56127, Italy; § Center for Nanotechnology Applied to Engineering (CNIS), Sapienza University of Rome, Rome 00185, Italy; ∥ Department of Basic and Applied Sciences for Engineering (SBAI), Sapienza University of Rome, Rome 00161, Italy

## Abstract

The advent of chimeric antigen receptor (CAR) T-cell therapy has introduced a novel and personalized approach to cancer treatment. Despite its promise, the challenge of developing a system that bypasses the need for viral vectors remains significant, particularly in terms of achieving a clinical efficacy and sustained durability. To address these challenges, lipid nanoparticles (LNPs) produced through advanced microfluidic technology have been recently utilized to encapsulate plasmid DNA (pDNA) encoding CAR receptors. However, the intrinsic challenges associated with pDNA encapsulation, along with the critical requirement for efficient expression, remain substantial obstacles. Here, we show that incorporating DNA-condensing agents into the microfluidic manufacturing of LNPs effectively overcomes these limitations. Briefly, we conducted a preliminary investigation to characterize LNPs with and without the commercial condensing agent P3000-Reagent (PR), focusing on their physicochemical properties and scrutinizing the biological outcomes primarily in the HEK-293 cell line. Our results demonstrated that precondensation of the pDNA with PR differentially increased the transfection efficiency of the tested formulations, whereas confocal microscopy indicated reduced lysosomal colocalization and major nuclear localization. Finally, PR was found to enhance LNP efficiency upon multiple administrations to the immortalized T-lymphocyte Jurkat cell line, enabling the delivery of both a luciferase reporter gene and a functional CAR-encoding plasmid. Overall, these findings underscore the great potential of introducing DNA-condensing agents into the LNP preparation process, especially for systems designed for challenging delivery applications, such as multiadministration transfection protocols.

## Introduction

Immunotherapy has significantly advanced cancer treatment, offering more personalized and targeted approaches. Among these, chimeric antigen receptor T-cell (CAR-T) therapy has shown remarkable promise, particularly in hematologic malignancies, and is now being explored for solid tumors.^1^ However, widespread application of this technology is still hindered by the reliance on viral vectors for gene delivery. While effective, viral-based approaches come with challenges such as high production costs, complex manufacturing, and potential risks of insertional mutagenesis.^2,3^ Herein, lipid nanoparticles (LNPs), produced through advanced microfluidic technology, have gained attention as promising nonviral delivery systems due to their biocompatibility, ability to encapsulate nucleic acids, and scalability for clinical applications.^4,5^ Particularly, LNPs have shown remarkable success mainly in the field of RNA delivery, as witnessed by the Food and Drug Administration (FDA) approval of Onpattro, in 2018 (encapsulating short interference RNA), for the treatment of polyneuropathies caused by hereditary transthyretin-mediated amyloidosis, and the recent mRNA-based vaccines for COVID-19.^6^ Nonetheless, RNA-delivery faces several hurdles principally arising from large-scale production and conservation. In addition, the majority of these systems provides administration via injection in the patients, which is closely tied to heightened mucosal immunogenicity.^7^ Given the need to overcome these barriers related to the RNA molecules, the delivery of plasmid DNA (pDNA) has gained traction during the last years, although lacking an equally extensive exploration. Indeed, the encapsulation of DNA molecules within a cationic lipid envelope offers several advantages, such as cost-effective large-scale production, enhanced thermostability, and improved ease of handling.^8,9^ Given these, the use of LNPs to deliver pDNA encoding CAR receptors represents an innovative approach, albeit not without challenges as the need to traverse both the cellular and nuclear membranes.^10^ To tackle this inherent limitation, we optimized a transfection protocol involving multiple administrations, which resulted in a marked improvement in gene transfer efficiency.^11^ Nevertheless, additional refinements are necessary to further enhance transfection outcomes. In this regard, an alternative strategy involves the incorporation of condensing agents into the manufacturing of LNPs. These agents facilitate the pDNA packaging, improving its encapsulation within LNPs, enhancing its stability, and promoting nuclear trafficking, thereby representing a promising avenue for advancing nonviral CAR-T engineering.^12–14^ A similar principle is employed in the formulation of Lipofectamine 3000, the current gold standard in lipofection, where pDNA is preincubated with specific reagents to optimize delivery efficiency. The protocol involves the mixing of the nucleic acid with the commercial P3000-Reagent (PR), whose composition remains undisclosed. However, it has been demonstrated that the omission of this preincubation resulted in reduced cellular transfection of the complex and higher cytotoxicity.^15^ In light of this context, this study first involved the preparation, characterization, and in vitro validation of two LNPs encapsulating PR–pDNA complexes, prepared by strictly adhering to the Lipofectamine 3000 protocol. Subsequently, by analyzing the subcellular localization and testing the previously mentioned multiple administration protocols in HEK-293 and Jurkat T cells, we found that precondensation with PR significantly improved the transfection efficiency (TE) of one formulation (i.e., PR–LNP2), reducing lysosomal degradation and increasing nuclear localization. In conclusion, our results demonstrate the advantages of multiadministration transfection protocols and suggest that the integration of condensing agents can further improve the efficiency of LNP-mediated pDNA delivery. Furthermore, this approach holds promise for optimizing nonviral gene transfer strategies, paving the way for more effective CAR-T engineering.

## Results

### Physical–Chemical Characterization

In this work, we investigated the potential effects of PR on the physicochemical properties of LNPs, as well as on cytotoxicity, TE, and intracellular behavior. As a preliminary step, the pDNA-condensing ability of PR was assessed in both water and acidic buffer (Figure S1), the latter being commonly used in LNP manufacturing to optimize interactions and facilitate pDNA encapsulation within the lipid envelope.^16^ Our results (detailed in the Supporting Information) clearly indicate that exposure of pDNA to PR led to monodisperse solutions of small-sized cationic complexes. Subsequently, both naked pDNA and PR–pDNA complexes were encapsulated into two distinct LNP formulations, designated LNP1 and LNP2, respectively. These formulations differ in their lipid compositions, as shown in Figure 1a and detailed in Table 1 (Materials and Methods). Briefly, both LNPs are PEGylated systems, with LNP1 being a balanced multicomponent mixture and LNP2 being enriched in cholesterol-derived lipids. After preparation, LNPs and PR–LNPs were characterized in terms of size and zeta potential by Dynamic Light Scattering (DLS). The corresponding distributions are reported in Figure 1b, along with the average values of size (Figure 1c), polydispersity index (PdI) (Figure 1d), and zeta potential (Figure 1e).

**Figure 1:**
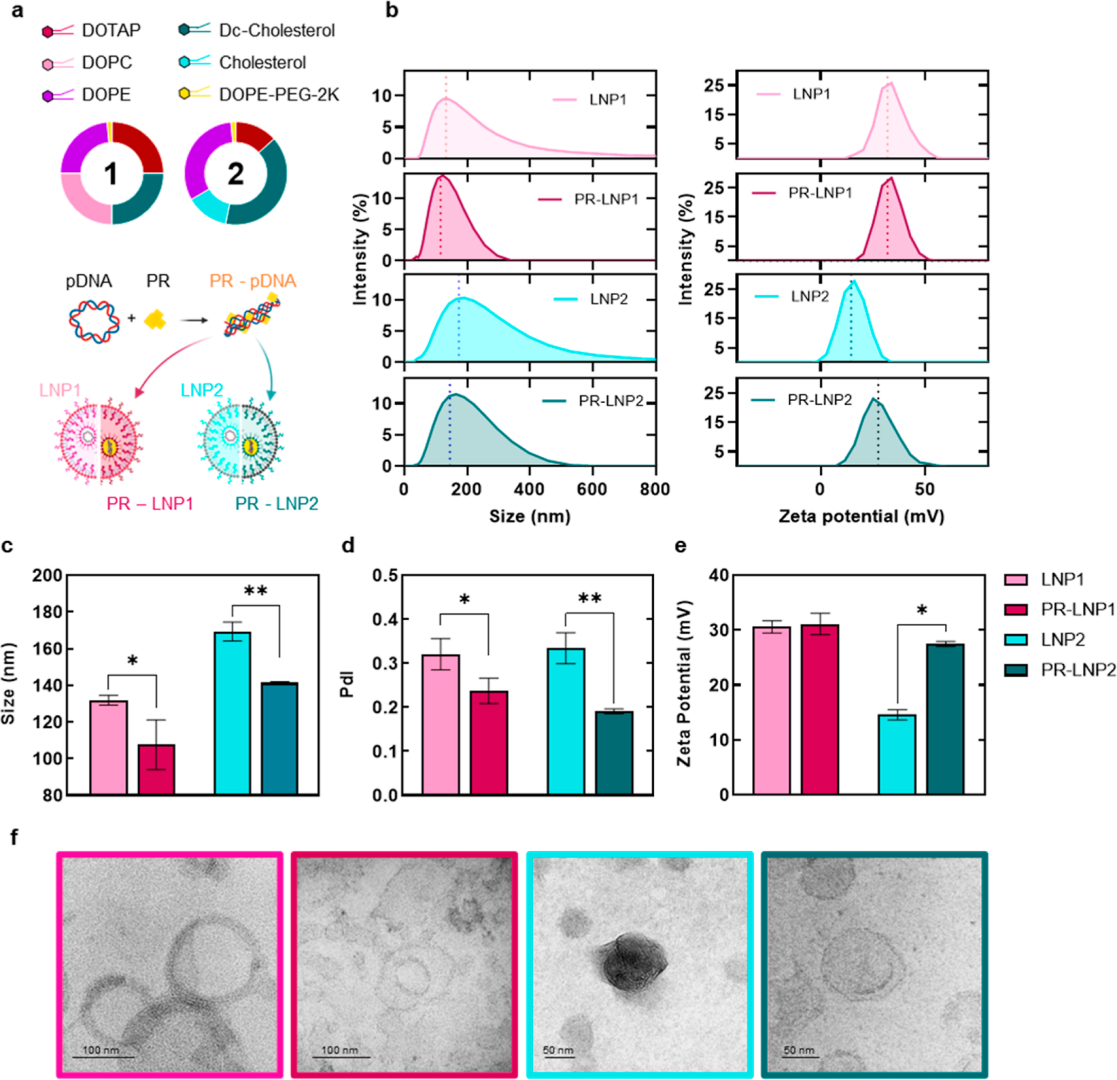
(a) Representative scheme of lipid mixtures (compositions depicted in the pie charts), pDNA, and PR that have been employed to prepare LNP1, PR–LNP1, LNP2, and PR–LNP2. (b) Size and zeta potential distributions of the systems. (c) Average size (Z-average), (d) polydispersity index, and (e) zeta potential of the investigated formulations. (f) Representative TEM images of LNPs and PR–LNPs. Statistical significance was evaluated using one-way ANOVA and Tukey’s multiple comparison test: *p < 0.05; **p < 0.01, ***p < 0.001, ****p < 0.0001. *N* = 3 technical replicates.

**Table 1:** Lipid Composition Expressed as the Molar Percentage of LNP1 and LNP2

ID	LNP1		LNP2	
	lipid	(%)	lipid	(%)
lipid composition	DOTAP	25	DOTAP	13.3
	DC-Chol	25	DC-Chol	39.9
	DOPE	23.5	DOPE	31.9
	DOPC	25	cholesterol	13.3
	DOPE-PEG 2000K	1.5	DOPE-PEG 2000K	1.5

Globally, the obtained outcomes from this physicochemical characterization indicate that preincubating pDNA with PR led to size reduction of the final LNP products, resulting in a size reduction from 169 to 141 nm for LNP1 and PR–LNP1, respectively, and from 132 to 108 nm for LNP2 and PR–LNP2 (Figure 1c), respectively. Additionally, this approach yielded more monodisperse solutions, with PdI values decreasing from 0.33 to 0.19 for LNP1 and PR–LNP1, respectively, and from 0.32 to 0.23 for LNP2 and PR–LNP2 (Figure 1d), respectively. LNP1 maintained its positive charge (i.e., about +30.5 mV) when PR was added, whereas LNP2, which was characterized by a low positive surface charge, displayed a remarkable increase of the zeta potential, from +14.6 to +27.5 mV (Figure 1e). Notably, a moderate surface charge increase makes the LNPs more prone to interact with the anionic cellular membrane and extracellular proteoglycans while maintaining the colloidal stability and reducing the aggregation phenomena of the particles.^17,18^ Finally, to gain deeper insights into the structural features of PR–LNPs and their corresponding controls, transmission electron microscopy (TEM) was performed. The representative images presented in Figure 1f reveal the variety of morphologies observed in the samples. LNPs, whose sizes align closely with the results from DLS analysis, displayed a well-ordered multilamellar nanostructure, which likely consists of alternating lipid bilayers with aqueous channels containing pDNA.^19,20^ In contrast, PR–LNPs exhibited a more swollen structure with reduced density in the core of the nanostructure, differing significantly from a multilamellar arrangement. Overall, the physicochemical characterization of the systems clearly revealed that PR ameliorated the synthetic features of LNPs by providing formulations with a smaller size, lower PdI, higher surface charge, and less-organized nanostructure.

### Transfection Efficiency and Cytotoxicity

The next step was to verify the TE of the formulations, intended as the capability of the systems (containing and not containing PR) to deliver the pDNA coding for the firefly luciferase protein (pMirGlo) to human embryonic kidney (HEK-293) cells, which represent an established model for in vitro transfection studies.^8,9,21–23^ Experiments were conducted to evaluate both the TE and the viability of the cells after the treatments. As shown in Figure 2a,b, PR significantly increased TE of the formulations without altering their biocompatibility at the administered dose and revealed a more remarkable impact on LNP2 compared to LNP1. Notably, the experiments were performed at two distinct incubation temperatures with the treatments, 37 and 4 °C, respectively, aiming to assess the influence of PR on the endocytic mechanism, which is a temperature-dependent process that predominantly influences the internalization of lipid-based systems.^24–27^ As reported in Figure 2a, TE values of LNP1 systems, both in the presence and absence of PR, were remarkably higher under physiological conditions (i.e., 37 °C) than those obtained at 4 °C. Furthermore, PR–LNP1 complexes exhibited slightly higher TE than the PR–lacking counterparts, whereas no temperature-dependent or PR-dependent significant effects were detected in cell viability (Figure 2b). This last trend was confirmed for LNP2 samples, which globally exhibited a TE significantly exceeding that of LNP1, as previously mentioned. Of note, PR remarkably increased the TE of LNP2 both at 37 and 4 °C conditions.

**Figure 2:**
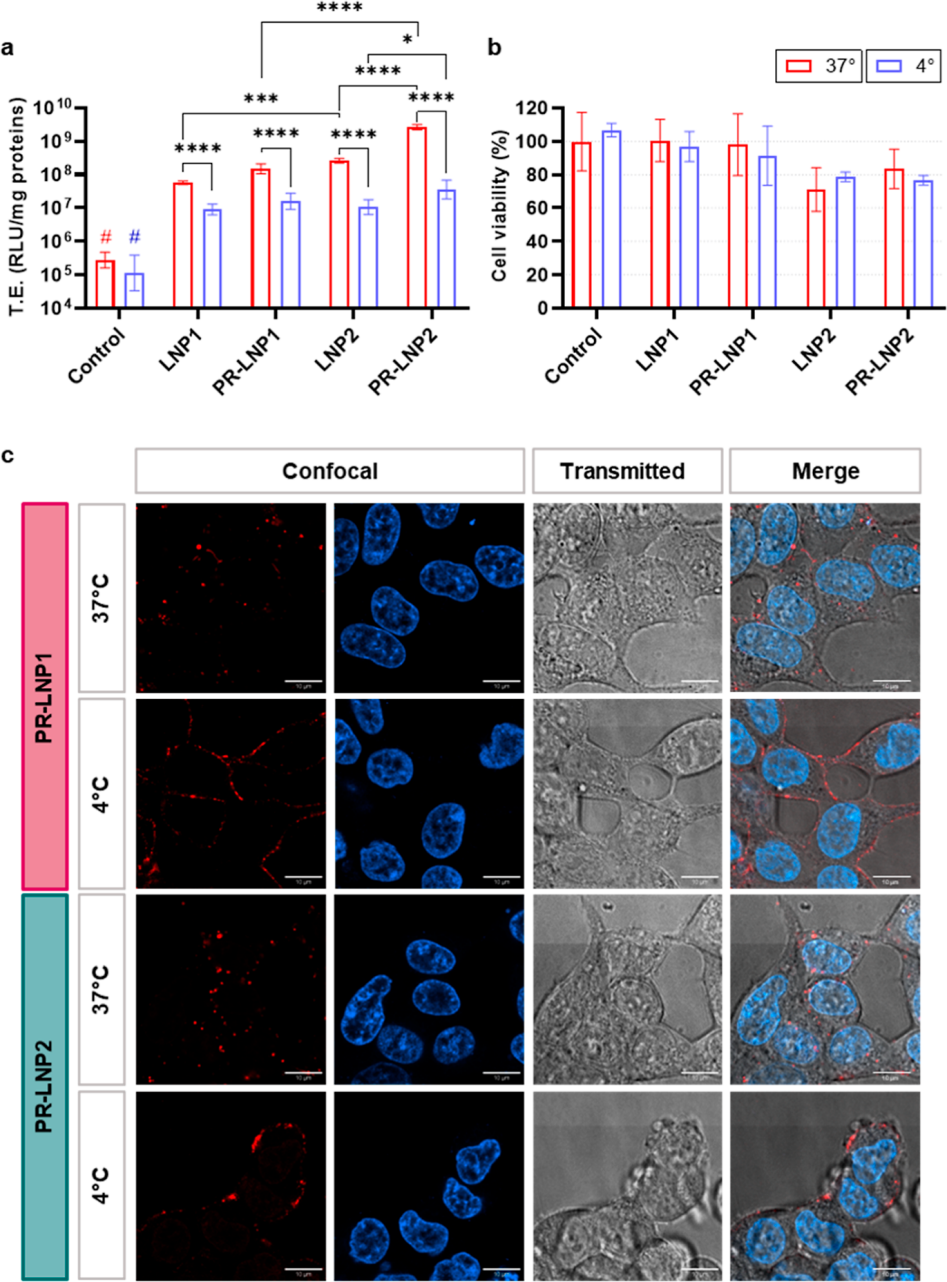
(a) TE of LNPs and PR–LNPs expressed as relative light units (RLUs)/mg of proteins. (b) Cell viability of HEK-293 cells 48 h after treatment expressed as a percentage with respect to untreated cells. (c) Confocal fluorescence microscopy of red-stained PR–LNPs both at 37 and 4 °C. Nucleus were blue-stained with Hoechst, particles were red-stained with Texas Red. Scale bar = 10 μm. Statistical significance was evaluated using two-way ANOVA and Tukey’s multiple comparison test: *p < 0.05; **p < 0.01, ***p < 0.001, ****p < 0.0001, *N* = 6 technical replicates for the TE experiment, *N* = 3 for the cell viability experiment.

To further interpret these findings, we conducted a confocal microscopy analysis aiming at investigating the uptake mechanism of PR–LNPs 2 h post-treatment and enabling a visual assessment of their intracellular localization. Figure 2c presents single-channel and merged images of HEK-293 cells treated under both 37 and 4 °C conditions. Although these images do not allow for precise quantification of PR–LNP uptake efficiency, a clear trend can be observed. Consistent with the transfection results, at 4 °C the red-stained PR–LNPs predominantly accumulated at the cell membrane, whereas at 37 °C, they exhibited a perinuclear distribution, as indicated by their proximity to the Hoechst-stained nuclei. This effect was particularly evident for PR–LNP2, suggesting a potential correlation with its higher TE observed. In this regard, a representative multichannel z-stack acquisition for PR–LNP2 is reported in the Supporting Information, Figure S2.

### Lysosomal Colocalization

We next investigated the colocalization of our treatments with lysosomes in HEK-293 cells (Figure 3). Using correlation analysis based on confocal microscopy images, we assessed that the addition of PR to LNP1 had no significant impact on the lysosomal degradation of the systems, as evidenced by the overlap of green and red signals in Figure 3a. The colocalization of these signals, representing lipids and lysosomal compartments, was quantified using Pearson’s and Mander’s M_2_ coefficient, which provides the proportion of the LNP signal that coincides with the lysosomal one (Figure 3b). In contrast, the analysis revealed a reduced tendency for lysosomal sequestration in the case of PR–LNP2, which notably differed from the pattern observed for LNP2.

**Figure 3:**
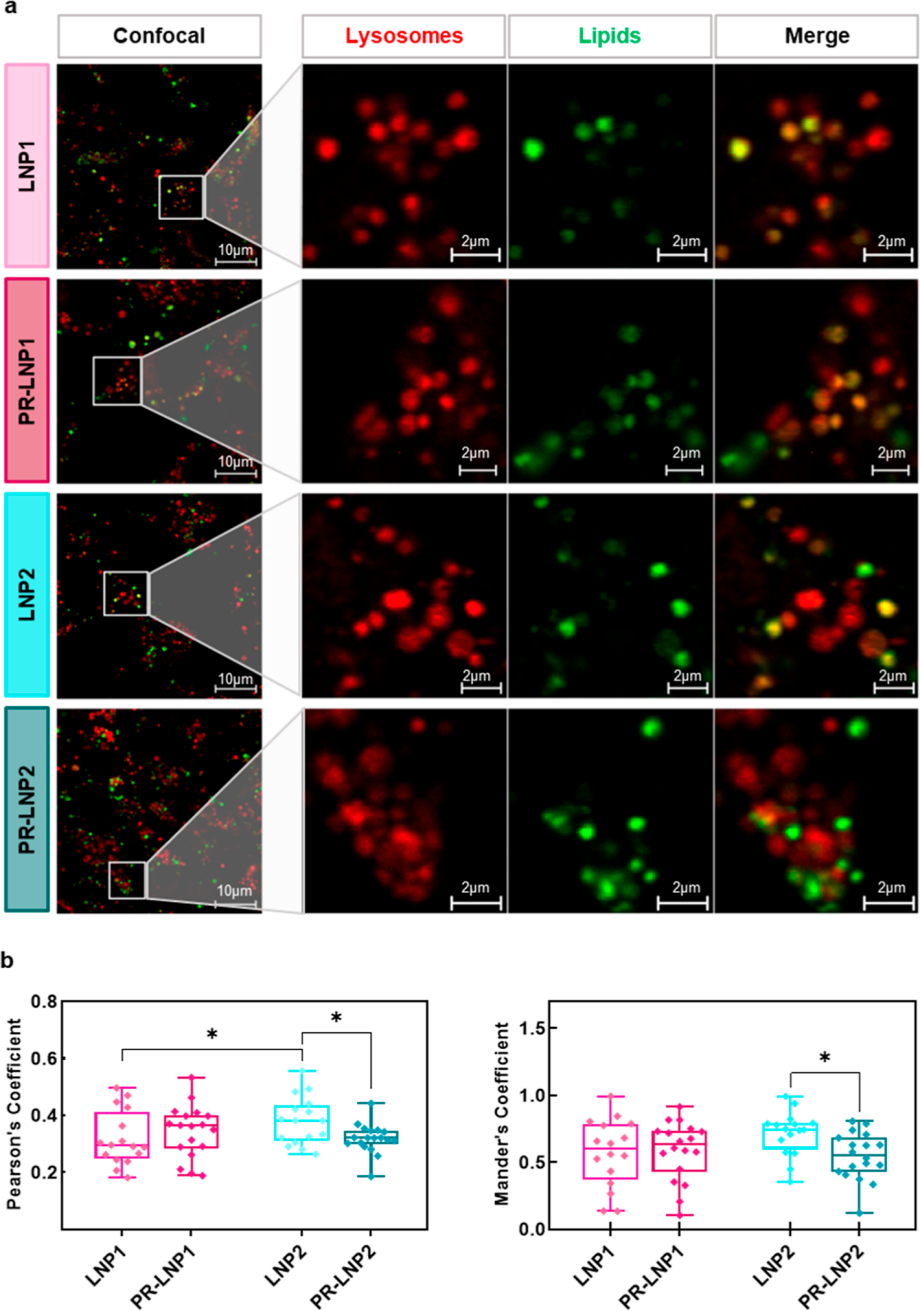
Colocalization with lysosomes of PR-LNPs and their control counterparts. (a) Representative confocal microscopy images where red and green channels indicate lysosomes and lipids, respectively. The quantification of colocalization has been made in terms of Pearson’s coefficient and Mander’s coefficient (b). Statistical significance was evaluated using one-way ANOVA and Tukey’s multiple comparison test: *p < 0.05; **p < 0.01, ***p < 0.001, ****p < 0.0001. *N* = 16 distinct images per class.

### Multiple Administration to the Jurkat Cell Line

As the last step of this work, we assessed whether the PR-induced improvement of LNP properties translated into enhanced TE in the immortalized T-lymphocyte Jurkat cell line, with the aim to better evaluate the impact of PR in a relevant cellular context. Based on the previously described outcomes from TE assays and confocal microscopy, we focused on the most effective formulation and its PR-lacking counterpart, i.e., PR–LNP2 and LNP2, respectively, and specifically evaluated the delivery efficiency by the Luciferase assay. The inherent challenge of transfecting Jurkat cells led us to adopt, in combination with the PR complexation, a well-optimized multiple administration protocol in order to implement and sustain an effective transfection during a prolonged time.^11,28^ For a detailed description of the protocol, please refer to the Materials and Methods section. Briefly, a portion of the cells was collected every 48 h for the assays, while the remaining cells were reseeded and retreated under the same conditions up to 5 times, referred to as “boost” (Figure 4a). This approach allowed us to monitor TE and cell viability over time while ensuring a continuous delivery of pDNA, as shown in Figure 4b,c. Results up to the third boost are presented here, while the complete set of data can be found in Figure S3 of the Supporting Information. Throughout a nine-day period, PR–LNP2 consistently exhibited significantly higher TE compared to its counterpart lacking precondensed pDNA. Overall, cell viability, ranging from 60% to 100%, did not show statistically significant differences, likely due to the considerable experimental variability. Prompted by the encouraging readouts, we repeated the protocol with the same treatments but encapsulating the CAR construct (pSLCAR-CD19) to assess the feasibility for a novel CAR-T-based protocols.^29^ Particularly, the lentiviral plasmid pSLCAR-CD19 has been designed to incorporate a P2A ribosomal self-skipping sequence separating the CAR construct from an enhanced green fluorescent protein (EGFP) marker, allowing CAR-expressing cells to become fluorescent and to be identified through FACS analysis.^30^ After adopting the same multiple-administration protocol, we achieved the following outcomes. Once again, PR–LNP2 proved to be a superior system with respect to LNP2, showing a significant increase in the number of GFP-positive cells but plateauing the transfection around the second boost (with a peak of approximately 13% GFP-positive cells). In this case, a significant reduction in cell viability was observed for LNP2 after the second boost, whereas PR–LNP2 maintained higher viability levels until a significant decrease emerged after the third boost. In contrast, LNP2 without PR reached similar transfection levels only by the third boost, and importantly, this achievement was coupled with a drastic reduction in cell viability.

**Figure 4:**
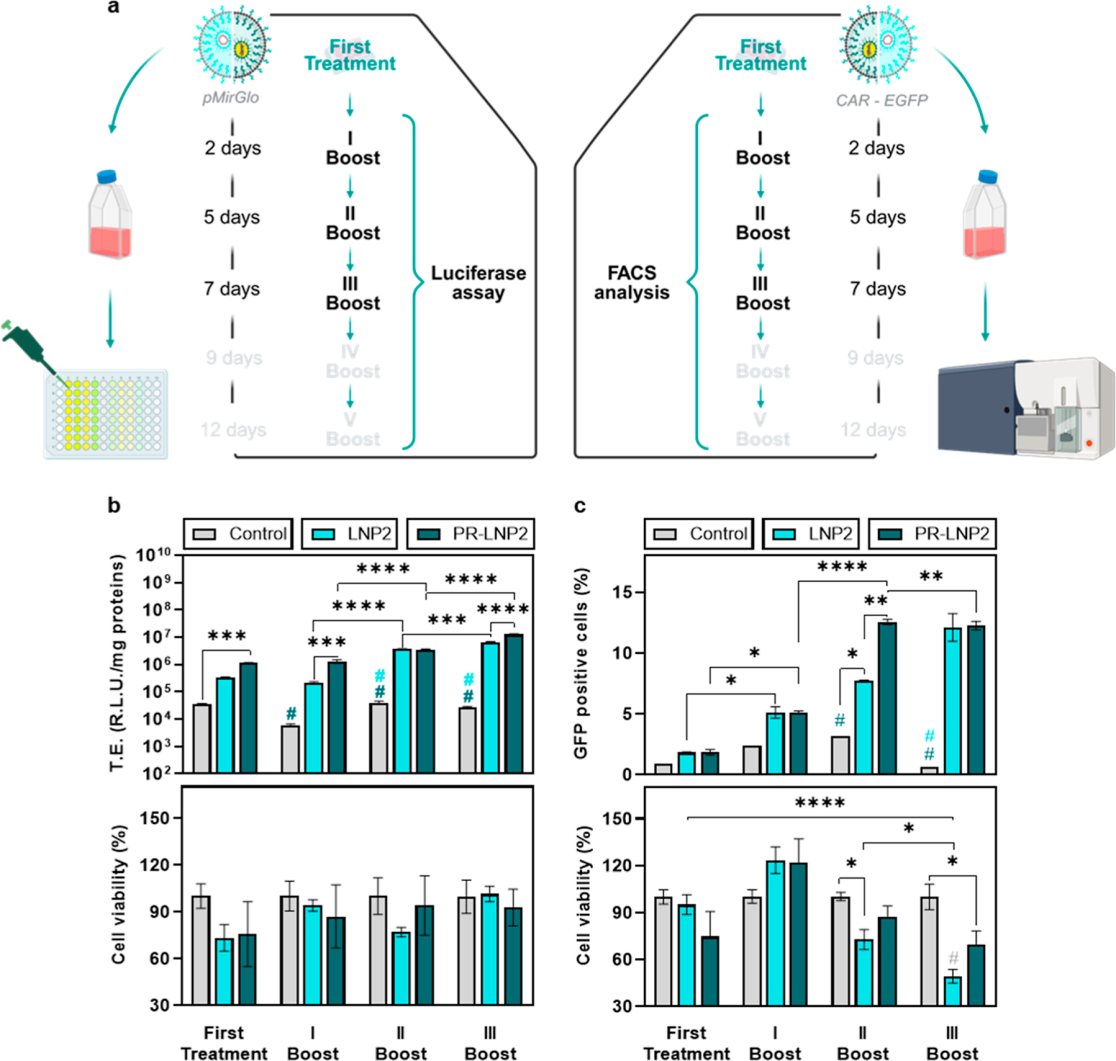
(a) Schematic representation of the experimental workflow. (b) TE of human leukemic Jurkat T cells during the multiple administration protocol. Cell viability of the human leukemic Jurkat cell line expressed as a percentage with respect to untreated cells. (c) TE of LNP2 and PR–LNP2, in terms of % GFP-positive cells of the Jurkat cell line. Statistical significance was evaluated using two-way ANOVA and Tukey’s multiple comparison test: *p < 0.05; **p < 0.01, ***p < 0.001, ****p < 0.0001, # indicates *p* < 0.0001 for control-involved comparison tests in the same boost. *N* = 6 technical replicates for the TE experiment, *N* = 3 for the cell viability experiment.

## Discussion

LNPs have gained considerable success as gene delivery systems due to their ability to efficiently encapsulate and deliver nucleic acids to cells. They have already been successfully used in clinical settings and have emerged as a promising alternative to viral vectors in cancer immunotherapies.^31,32^ As an instance, it has been demonstrated that LNPs can effectively deliver mRNA encoding CARs to primary human T cells, achieving comparable TE to electroporation while exhibiting reduced toxicity.^33,34^ In the context of ionizable LNPs, pDNA has also been successfully employed to achieve an effective delivery of CARs to both Jurkat and primary T cells.^35^ As an alternative to these conventional LNPs, in this work, we explore the use of LNPs containing stable cationic lipids and investigate the effects of incorporating PR as a pDNA-condensing agent during the preparation process. Specifically, we aimed at preparing, characterizing, and validating LNP formulations encapsulating a precondensed pDNA encoding for a CAR receptor. The initial stage of the study included the choice of lipid compositions that served for the LNP synthesis through a state-of-the-art microfluidic process. In particular, we draw from the insights gained in a previous work conducted within our group, where we focused on a screening procedure for the optimization of a multicomponent pDNA–LNP library varying in lipid composition, surface functionalization (i.e., PEGylation), and influential manufacturing factors of microfluidic LNP preparation (i.e., total flow rate (TFR), flow rate ratio (FRR), and amine to phosphate (N/P) ratio).^8^ Herein, we chose two systems referred to as LNP1 and LNP2, both primarily comprised the same cationic lipids, 1,2-dioleoyl-3-trimethylammonium-propane (DOTAP) and 3β-[*N*-(*N*′,*N*′-dimethylaminoethane)-carbamoyl]-cholesterol (DC-Chol), both pivotal for their electrostatic interaction with negatively charged pDNA.^36^ Additionally, the formulations included the same PEGylated lipid, 1,2-dioleoyl-*sn*-glycero-3-phosphoethanolamine-*N*-[amino (polyethylene glycol)-2000] (DOPE-PEG2000), commonly used to increase the biocompatibility and to avoid the interactions between particles by creating a steric hindrance on the particle surface.^8^ So, the key distinction lied in the composition of the remaining helper lipids, generally crucial for the stability of the nanoparticle structure.^37^ While both systems contained dioleoyl-phosphatidylethanolamine (DOPE), LNP1 uniquely featured 1,2-dioleoyl-*sn*-glycero-3-phosphocholine (DOPC), whereas LNP2 contained cholesterol, known as a promoter of the membrane fusion and gene transfer at a specific concentration.^38^ Following the identification of optimal lipid compositions, we directed our focus to the precomplexation of pDNA with the PR compound prior to microfluidic formulation. This strategy notably influenced the physicochemical properties of the resulting LNP systems. In particular, the introduction of PR generally yielded more monodisperse formulations, and in the case of LNP2, enriched with a higher content of cholesterol-like lipids, the precondensation process also led to a marked decrease in the particle size. Overall, these outcomes provide strong evidence of the condensing effect of PR when incubated with pDNA, aligning its unknown chemical composition with those of other well-known condensing agents^17^ (i.e., Protamine Sulfate,^18^ Chitosan,^19^ or poly-l-lysine^20^). Furthermore, the size reduction is particularly advantageous, given that smaller complexes are more readily taken up and processed by cells.^39^ Therefore, the ability of PR complexation to modulate these key parameters without the need for additional physical processing steps, such as filtration or extrusion, offers a compelling and scalable alternative for improving the LNP physical–chemical properties. Results from in vitro validation clearly demonstrated that our formulations enriched in cholesterol-based lipids (i.e., LNP2 and PR–LNP2) were markedly more efficient than their counterparts, further confirming the central role of cholesterol in LNP-mediated gene delivery. Indeed, previous studies on other lipid-based nanoparticles have explored how cholesterol acts also to reduce metabolic degradation and positively influence interactions between lipid-based systems and cellular membranes. These effects result in a favorable internalization and increased endosomal escape.^40,41^ Herein, to further elucidate the temperature-dependent internalization of our LNP formulations, we conducted experiments at both 37 and 4 °C and complemented these studies with confocal microscopy. At 37 °C, the confocal images clearly showed that the nanoparticles, regardless of PR complexation, reached a perinuclear location, indicative of efficient endocytic uptake and subsequent intracellular trafficking. In contrast, at 4 °C, the particles remained largely confined to the cell membrane, corresponding with a significant reduction of the transfection performances. These integrated observations indicate that the basic cellular processes governing nanoparticle uptake remain largely unaltered by the addition of PR, with the endocytosis that remains the primary route of entry.^42^ Therefore, the enhanced performance of PR–LNP2 appears to arise from factors downstream of internalization rather than modifications of the uptake mechanisms themselves. Indeed, once internalized, LNPs must overcome the critical barrier of endosomal escape to reach the cytosol before undergoing degradation. The endosomal compartment progresses through distinct maturation stages, ultimately leading to lysosomal formation, where the LNP accumulation represents the most significant bottleneck to their efficiency and applicability.^43,44^ Our colocalization analysis further supports previous observations. Specifically, the data indicate that PR may enhance the endosomal escape of the cholesterol-enriched formulation, resulting in reduced lysosomal sequestration. This reduction in lysosomal accumulation likely contributes to the superior TE observed for PR–LNP2, confirming that the beneficial effects of PR are formulation-dependent, possibly due to inherent differences in lipid composition and electrostatic interactions. In other words, the observed differences in colocalization patterns with lysosomes are likely due to the distinct compositions of the investigated formulations and are consistent with previous studies reporting lower lysosomal degradation, more efficient endosomal escape, and higher transfection efficiency in cholesterol-enriched systems.^40,41^ However, it should be noted that the precise mechanism of action of PR remains unclear and may involve formulation-dependent changes in lipid organization, as suggested by the TEM images showing more pronounced structural modifications in the cholesterol-enriched LNP. Finally, to test the applicability of PR–LNPs in a more complex cellular context, we extended our investigations to the Jurkat cell line, which is notoriously difficult to transfect and serves as a critical model for studying T-cell receptor activation and the associated signaling pathways.^45,46^ In this final phase of the study, we paired the luciferase assay (also used in the previous validation on HEK-293 cells) with GFP expression to obtain a more informative picture in terms of the percentage of transfected cells. Of note, we applied a multiple administration protocol validated in our laboratory, which has previously shown to achieve sustained transient expression without compromising cell viability.^11,28^ Using repeated pDNA delivery every 48 h, we conducted the experiments and identified the third boost as the optimal end point for analysis. This limited duration was deliberately chosen, as previous studies have shown that extending in vitro expansion beyond 2 weeks can lead to the upregulation of exhaustion markers in T cells, ultimately altering their phenotype.^47^ When combined with PR complexation, our approach consistently resulted in higher luciferase activity and a significantly increased proportion of GFP-positive cells using a CAR construct (pSLCAR-CD19) with a P2A-linked EGFP marker. With PR complexation, TE peaked approximately by the second boost while maintaining robust cell viability throughout the experiment. Conversely, LNP2 without PR reached similar transfection levels only with the third boost but was remarkably more cytotoxic. As LNP2 and PR–LNP2 shared the same lipid composition and exhibited similar physicochemical properties, we speculate that the observed differences in LNP cytotoxicity were cargo-dependent. This may be due to differences in lipid shell organization, which in turn could lead to variations in cellular uptake, membrane interactions, and intracellular trafficking, eventually causing cumulative cytotoxicity upon repeated dosing. In this context, a further cargo-dependent effect on LNP cytotoxicity after multiple doses was evident. Indeed, Jurkat cells transfected with formulations carrying the CAR-T plasmid exhibited a more pronounced viability loss at the third boost compared to those transfected with luciferase-encoding pDNA, possibly due to differences in plasmid size or expression burden.

## Conclusions

In conclusion, our study demonstrates that incorporating PR as a condensing agent in pDNA-loaded LNP formulations significantly improves the chemical–physical properties of the systems, resulting in enhanced in vitro delivery efficiency, particularly for cholesterol-enriched LNPs. Enhanced intracellular trafficking, marked by increased nuclear proximity, and reduced lysosomal degradation appear to be driving factors. Furthermore, our findings reveal that PR not only boosts the TE of LNPs in a model cell line (i.e., HEK-293 cells) but also overcame the inherent challenges in transfecting Jurkat T cells, which represents a critical model for CAR-T engineering and research. Notably, the application of a multiple administration protocol with CAR-encoding LNPs led to an increased proportion of GFP-positive cells, emphasizing the potential of this strategy in enhancing the generation of functional CAR-T cells. Taken together, our findings highlight the value of integrating DNA-condensing agents into the LNP formulation process, offering a strategic advantage in the rational design of more effective and versatile delivery platforms. This may be particularly relevant for demanding applications such as the scalable development of CAR-T cell therapies.

## Material and Methods

### Chemicals

The zwitterionic lipids dioleoylphosphatidylethanolamine (DOPE), 1,2-distearoyl-*sn*-glycero-3-phosphocholine (DSPC), and dioleoylphosphocholine (DOPC), along with cholesterol (Chol), 1,2-distearoyl-*sn*-glycero-3-phosphoethanolamine-*N*-[amino­(polyethylene glycol)-2000] (DSPE-PEG(2000)), and the cationic lipids 1,2-dioleoyl-3-trimethylammoniumpropane (DOTAP) and (3β-[*N*-(*N*′,*N*′-dimethylaminoethane)-carbamoyl])-cholesterol (DC-Chol), were purchased from Avanti Polar Lipids (Alabaster, AL). Texas red-DOPE (Life Technologies, Carlsbad, CA) was purchased from Sigma-Aldrich, Inc. (Merk KGaA, Darmstadt, Germany). Lipids were used without an additional modification. The pDNA encoding the firefly luciferase reporter gene (pmirGLO) was bought from Promega (Madison, WI, USA). The pDNA coding for the CAR construct (pSLCAR-CD19-28z) was a gift from Scott McComb (Addgene plasmid #135991; http://n2t.net/addgene:135991; RRID:Addgene_135991).

### Cell Culture

The human embryonic kidney-293 (HEK-293) cell line was purchased from the American Type Culture Collection (ATCC, Rockville, MD, USA) and maintained in culture in Dulbecco’s modified essential medium (DMEM, Gibco, Life Technologies, Carlsbad, CA, USA) enriched with 10% fetal bovine serum (FBS, Gibco, Life Technologies) and 1% penicillin–streptomycin (Gibco, Life Technologies). Cells were maintained at 37 °C with 5% CO_2_ under a humidified atmosphere.

Jurkat cell lines were purchased from the American Type Culture Collection (ATCC, Rockville, MD, USA) and maintained in the RPMI-1640 (Gibco, Life Technologies, Carlsbad, CA) medium supplemented with 2 mM l-glutamine, 100 IU/mL penicillin/streptomycin (Gibco, Life Technologies), and 10% fetal bovine serum (FBS, Gibco, Life Technologies).

### Microfluidic Preparation of LNPs and PR–LNPs

Individual lipid stocks were prepared by dissolving cationic lipids DOTAP and DC-Chol, zwitterionic lipid DOPE or DOPC, cholesterol, and the PEG-lipid, DOPE-PEG 2000, in absolute ethanol to have a final total concentration of 6.25 μM. The lipid composition expressed as molar percentage of LNP1 and LNP2 is shown in Table 1.

Regarding the pDNA, 420 micrograms were solved in 25 mM sodium acetate buffer (AB) solution (pH = 4). For the PR–LNP synthesis, pDNA was precomplexed with the P3000 Reagent (PR) by pipetting. PR was obtained from the commercial kit of the Lipofectamine 3000 (Life Technologies, Carlsbad, CA, USA) and used for the complexation following the ratio of 2 μL/μg pDNA, as indicated in the commercial kit protocol. All of the LNPs were characterized by the final concentration of 0.1 mg/mL of pDNA. LNPs were finally obtained using a Y-shape staggered herringbone micromixer (SHM) (NanoAssemblr Benchtop from Precision NanoSystems Inc., Vancouver, BC, Canada), which involves the mixing at the Y-junction of the pDNA or pDNA + PR in AB and the lipids in ethanol solution at a flow rate ratio of 3:1 (AB to ethanol), at room temperature.

The two formulations were synthesized at the same DNA/lipid weight ratio (Rw = 10), which corresponds to a nitrogen to phosphate charge ratio (N/P) of 3, where the nitrogen derives from the cationic lipid and the phosphate from the nucleic acid. LNPs and PR–LNPs were subsequently dialyzed for 19 h against 500 mL of phosphate-buffered saline (PBS) at pH 7.4 with Slide-A-Lyzer Dialysis cassettes (0.5–3 mL, MWCO 3 kDa, Thermo Scientific, Rockford, MI, USA), to remove the residual ethanol.

### Characterization of LNPs

Particle size, polydispersity index, and zeta-potential were measured by DLS at 25 °C using a Zetasizer Nano ZS90 (Malvern, U.K.). The measurements of LNPs and PR–LNPs were carried out by diluting the sample 1:10 with distilled water, and the results were reported as mean ± standard deviation of three independent replicates. Regarding the measurements of pDNA and PR, we used 30 μL of each one and added 70 μL of distilled water or AB, in order to have a less diluted sample to ameliorate the detection of the instruments.

### Transmission Electron Microscopy

For TEM analysis, samples were diluted to 1 mg/mL in ultrapure water and applied to Formvar/carbon-coated copper grids (Ted Pella 01801) that had been glow-discharged for 120 s using a Pelco easiGlow unit. The grids were then negatively stained with 2% uranyl acetate, gently rinsed with ultrapure water, and air-dried. Imaging was conducted on a JEOL JEM-F200 transmission electron microscope operating at an accelerating voltage of 200 kV. Micrographs were acquired by using a GATAN Rio 16 CMOS camera.

### TE Assay on HEK-293

Biological evaluation of LNPs and PR–LNPs was assessed by an in vitro experiment on human embryonic kidney 293 (HEK-293) cells (ATCC, Rockville, MD, USA). Cells were grown in DMEM supplemented with 10% FBS. For the transfection experiments of HEK-293, cells were seeded on 24-well plates (40,000 cells/well) and then treated for 3 h in 400 μL Opti-MEM medium (Life Technologies, Carlsbad, CA, USA) with LNPs or PR–LNPs with 1 μg DNA/well (for the 1× condition). During the 3 h, the cells were subjected at the controlled temperatures of 37 or 4 °C. Each treatment has been performed in triplicate, and the Lipofectamine 3000 was used as the positive control at a 1× DNA condition following the standardized protocol (Life Technologies, Carlsbad, CA, USA). Then, after the 3 h, DMEM 20% FBS was added for HEK-293. After 48 h, luciferase expression of cells was measured by means of a Luciferase Assay System (Promega, Madison, WI, USA). Successively, cells were washed in phosphate saline buffer and 80 μL of lysis buffer 1× (Promega) was added in each well. Then, 10 μL of the cell suspension was diluted with 100 μL of the luciferase substrate (Promega) and the remaining 10 μL was used for the BCA assay. The TE expressed was determined by a Pierce BCA Assay Protein Kit (Thermo Fisher Scientific, Waltham, MA, USA) and expressed as Log_10_ of the Relative Light Units (RLUs) per milligram of cell proteins.

### Cell Viability Assay

Cell viability of HEK-293 cells was evaluated after the 48 h-treatment by 2,3-Bis­(2-Methoxy-4-Nitro-5-Sulfophenyl)-2*H*-Tetrazolium-5-Carboxanilide (XTT assay, cell proliferation Kit II, Roche). Cells were seeded on 96-well plates (10,000 cells/well) and transfected as explained in the TE Assay on HEK-293 “Transfection efficiency assay” section. After 48 h of incubation, 50 μL of XTT solution, prepared as indicated in the kit protocol, was added to each well and cells were incubated at 37 °C for 3 h. After that, the absorbance of each well was measured with a GloMax Discover System (Promega, Madison, WI, USA).

### Confocal Microscopy

Live-cell imaging was performed using a Zeiss LSM 800 confocal microscope equipped with a 63×/1.4 N.A. oil immersion objective and GaAsP detectors. HEK-293 cells (approximately 2 × 10^5^) were seeded in 22 mm WillCo glass-bottom dishes about 24 h prior to imaging. On the day of the experiment, cells were incubated with Texas Red-labeled LNPs for 2–3 h at 37 °C. For lysosome and DNA staining, cells were incubated with LysoTracker Deep Red (Thermo Fisher) for 30 min or Hoechst 33342 (Thermo Fisher) for 20 min before imaging. Excitation/emission was as follows: Hoechst was excited at 405 nm with emission collected between 410 and 550 nm; Texas Red was excited at 561 nm, and the emission was collected in the 570–630 nm range; LysoTracker Deep Red was excited at 633 nm with emission collected in the 650–750 nm range. To assess the colocalization between LNPs and lysosomes, Manders’ and Pearson correlation coefficients were calculated using the JaCoP plugin for ImageJ software.

### Transfection Efficiency in the Jurkat Cell Line

A deep in vitro validation of the PR–LNP2 was achieved by transfection experiments on the Jurkat cell line, both with pMirGlo and pSLCAR-CD19 pDNAs. Cells were seeded on a 12-well plate (500,000 cells/well) and treated with PR–LNP2 and LNP2. Every 48 h, cells were collected and reseeded before undergoing the same transfection procedure, which was repeated up to 5 times. Cells were then incubated for 48 h at 37 °C and 5% CO_2_. For the Luciferase Assay, cells were washed in PBS 1× (phosphate-buffered saline) and lysed using lysis buffer 1× 300 μL/well. Then, 30 μL of the cell lysate was placed in three wells of a white Corning 96-well solid polystyrene microplate (Sigma-Aldrich, Milan, Italy). The 10 μL present in each well was diluted with 100 μL/well of the luciferase substrate (Promega), while the remaining 30 μL was divided into three wells (10 μL/well) and used for the BCA assay. The TE is expressed as relative light units per milligram of cell proteins, while the protein amount was determined by the Pierce BCA assay protein kit (Thermo Fisher Scientific, Waltham, MA, USA). For the evaluation of the effective delivery of the pSLCAR-CD19 pDNA, cells were washed in PBS 1× (phosphate-buffer saline). GFP expression was measured after 48 h of each treatment through FACS analysis using a FACSCanto flow cytometer (BD Biosciences, San Jose, CA) and measured as the percentage of positive cells by the gating procedure. The data analysis was performed using the FlowJo program.

## Supplementary Material



## Notes

Condensing effect of PR on pDNA through reduced size and polydispersity and a zeta potential shift, nuclear localization of PR–LNP2-delivered DNA in HEK-293 cells by confocal microscopy, and PR–LNP2 improves TE in Jurkat T cells upon repeated administration without affecting cell viability (PDF)
