# Supplementary material for: Precondensed Plasmid DNA Enhances CAR‑T Cell Generation via Lipid Nanoparticles
Source: ACS Omega. 2025 Jul 4;10(27):29804–14. doi: 10.1021/acsomega.5c04308 (PMC12268738; doi:10.1021/acsomega.5c04308)
Supplement: Supplementary file 1 [file ao5c04308_si_001.pdf]

Pre-condensed plasmid DNA Enhances CAR-T Cell Generation via Lipid Nanoparticles

Andrea Pirrottina,[1#] Serena Renzi,[1#] Luca Digiacomo,[1] Francesca Giulimondi,[1] Valentina De Lorenzi,[2] Samuele Ghignoli,[2] Luca Pesce,[2] Francesco Cardarelli,[2] Francesco Mura,[3] Giacomo Parisi,[3] Luca Buccini,[4] Chiara Cassone,[1] Alessandra Zingoni,[1,3] Daniela Pozzi,[1,3] Giulio Caracciolo[1,3]*

[1]Department of Molecular Medicine, Sapienza University of Rome, 00161 Rome, Italy

[2]Laboratorio NEST, Scuola Normale Superiore, 56127 Pisa, Italy

[3]Center for Nanotechnology Applied to Engineering (CNIS), Sapienza University of Rome, 00185, Rome, Italy

[4]Department of Basic and Applied Sciences for Engineering (SBAI), Sapienza University of Rome, 00161, Rome, Italy

# equal contribution

Corresponding author: Giulio Caracciolo; Email: giulio.caracciolo@uniroma1.it

Keywords: DNA condensing agent; Lipid Nanoparticles; CAR-T; transfection efficiency

1. Physical-Chemical characterization of pDNA and PR-DNA

Dynamic Light Scattering (DLS) measurements allowed us to track the changes in size, polydispersity index (PdI), and zeta potential of the pDNA. We conducted this assessment both before and after the addition of PR, following the guidelines and ratios outlined in the Lipofectamine 3000 protocol. When PR was employed, as illustrated in Figure **S1**, size analysis in water (H_2O) revealed a reduction in size from 870 nm to 128 nm and polydispersity index (PdI) from 0.78 to 0.22, while the initially anionic surface of pDNA (i.e., zeta potential = -23.5 mV) shifted towards cationic values (i.e., zeta potential = 37.4 mV). To closely replicate the conditions of microfluidic synthesis, we also performed measurements in an acidic aqueous buffer (AB), which is commonly used in LNP manufacturing to optimize interactions and facilitate DNA encapsulation within the lipid envelope [16]. In this case as well, PR induced a reduction in size from 665 nm to 106 nm, consequently decreasing also the PdI from 0.9 to 0.2 and inducing a shift towards a cationic surface (i.e., from zeta potential = -18.7 mV to 20.8 mV). These outcomes provide strong evidence of the condensing effect of PR when incubated with pDNA, aligning its unknown chemical composition with those of well-known condensing agents [17] (i.e., Protamine Sulfate [18], Chitosan [19], or poly-L-lysine [20]).

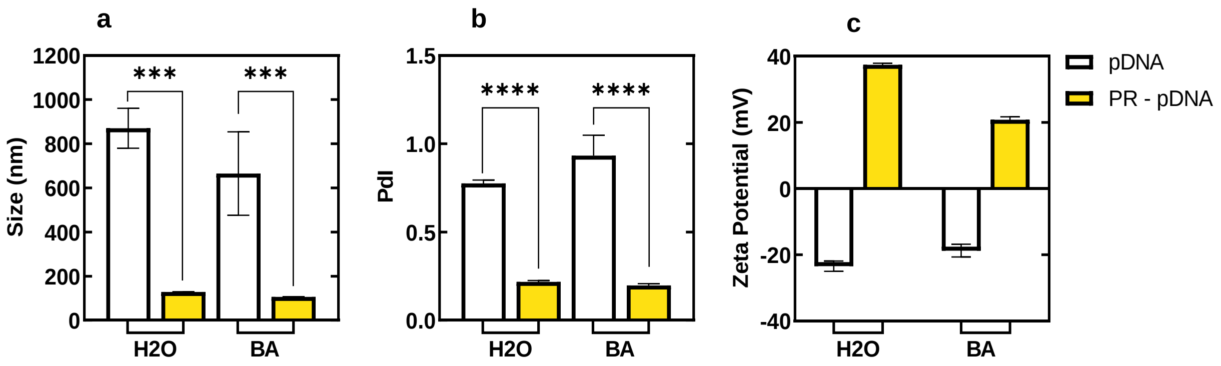

Figure S1. Physical-Chemical characterization of pDNA and PR-DNA in H2O and Acidic aqueous buffer (BA). (a) Size, (b) polydispersity index (PdI) and (c) zeta potential (mV). Statistical significance was evaluated using One-way ANOVA and Tukey's multiple comparison test: *$p < 0.05$; ** $p < 0.01$, *** $p < 0.001$, **** $p < 0.0001$.

2. Intracellular localization of PR-LNP2

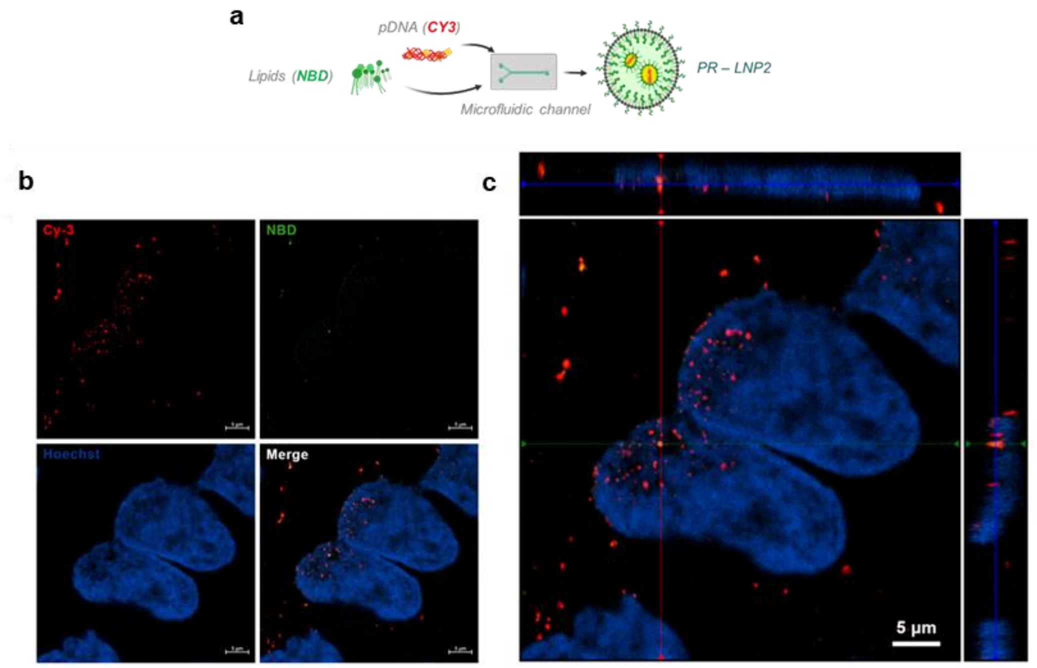

Figure S2. (a) Schematic illustration of the fluorescent marker: to visualize intracellular localization, PR-LNP2 was synthesized using NBD-labelled lipids (green) and encapsulating CY3-labeled PR-condensed pDNA (red). **(b)** Confocal microscopy images of HEK-293 after the transfection with CY3-labeled plasmid encapsulating PR – LNP2. The images, and in particular the z-stack acquisitions **(c)**, showed the nuclear colocalization of the transfected CY3-labeled plasmid 3 hours after the transfection experiment.

3. Impact of Repeated Lipid Nanoparticle Administration on Jurkat T Cell Transfection and Viability

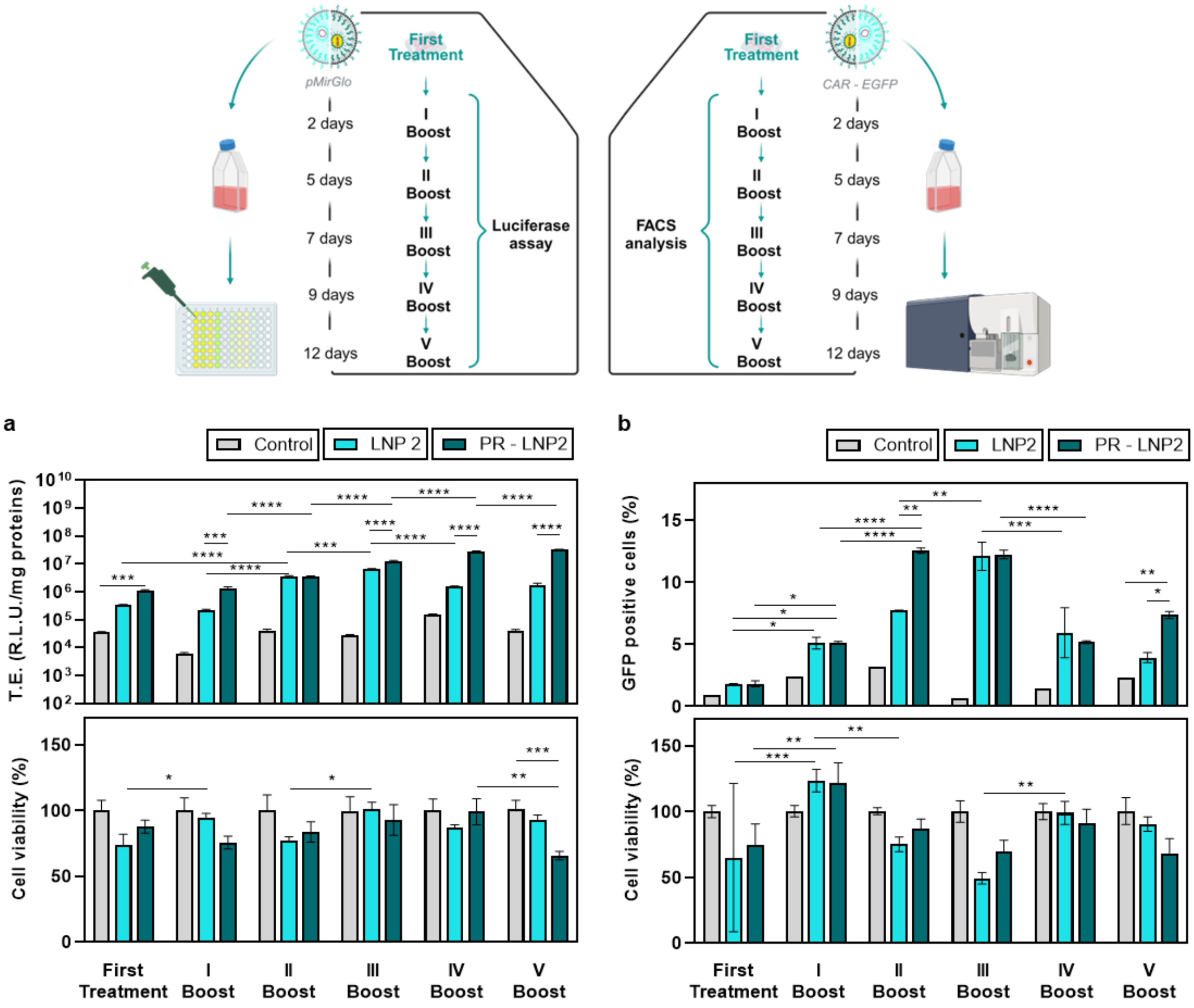

Figure S3. (a) TE and cell viability of human leukemic Jurkat T cells during the multiple administration protocol. (b) TE of LNP2 and PR – LNP2, in terms of % GFP-positive cells of the Jurkat cell line. Statistical significance was evaluated using Two-way ANOVA and Tukey's multiple comparison test. ****$p < 0.0001$.
